# Role of Translationally Controlled Tumor Protein in Cancer Progression

**DOI:** 10.1155/2012/369384

**Published:** 2012-04-17

**Authors:** Tim Hon Man Chan, Leilei Chen, Xin-Yuan Guan

**Affiliations:** ^1^Department of Clinical Oncology, Li Ka Shing Faculty of Medicine, The University of Hong Kong, Hong Kong; ^2^State Key Laboratory of Liver Research, The University of Hong Kong, Hong Kong; ^3^State Key Laboratory of Oncology in Southern China, Sun Yat-sen University Cancer Center, Guangzhou 510060, China

## Abstract

Translationally controlled tumor protein (TCTP) is a highly conserved and ubiquitously expressed protein in all eukaryotes—highlighting its important functions in the cell. Previous studies revealed that TCTP is implicated in many biological processes, including cell growth, tumor reversion, and induction of pluripotent stem cell. A recent study on the solution structure from fission yeast orthologue classifies TCTP under a family of small chaperone proteins. There is growing evidence in the literature that TCTP is a multifunctional protein and exerts its biological activity at the extracellular and intracellular levels. Although TCTP is not a tumor-specific protein, our research group, among several others, focused on the role(s) of TCTP in cancer progression. In this paper, we will summarize the current scientific knowledge of TCTP in different aspects, and the precise oncogenic mechanisms of TCTP will be discussed in detail.

## 1. Introduction

Translationally controlled tumor protein (TCTP) is a highly conserved multifunctional protein. Since the discovery of TCTP over two decades ago, the expression level of TCTP has been investigated in more than 500 tissues and cell types. Expression levels have been found to vary by nearly two orders of magnitude between different types of tissues [1] with preferential expression in mitotically active tissues [2]. Two mRNA transcripts have been reported to carry the same 5′UTR but different 3′UTR using alternative polyadenylation signals. The solution structure of TCTP from fission yeast also revealed structural similarity with the Mss4/Dss4 protein family. The expression of TCTP is highly regulated both at transcriptional and translational levels in addition to a wide range of extracellular signals. It has been implicated in many cellular processes, such as cell growth, cell cycle progression, apoptosis, malignant transformation, and the regulation of pluripotency. Although TCTP is not a tumor-specific protein, the downregulation of TCTP was found in tumor reversion [3]. Our research group and others also substantiated the link between TCTP deregulation and cancer progression [4–6].

This paper will mainly focus on the biological functions of TCTP and malignant transformation induced by TCTP. Furthermore, the clinical implications of TCTP in human cancers will also be discussed.

## 2. Features of TCTP mRNA and Protein

### 2.1. TCTP mRNA

The human TPT1 gene coding for TCTP spanning about 4.2 kb, consists of six exons and five introns [7]. Two alternative polyadenylation signals at 3′UTR generated two mRNA transcripts that differ in the length of 3′UTR. In all mammalian tissues tested, both types of mRNAs are expressed at different ratios, and the shorter transcript is usually expressed more abundantly [2]. Sequence analyses of TCTP transcripts indicated that the 5′UTR is CG-rich with a high degree of secondary structure. TCTP mRNA has a 5′ terminal oligopyrimidine tract (5′TOP), which is a signature of translationally controlled mRNAs [8]. Although AU-rich regions and AUUA elements have been identified at the 3′UTR, they do not match classical mRNA instability elements [1].

### 2.2. TCTP Protein

As revealed by sequence alignment of TCTP in more than 30 different species, TCTP is highly conserved over a long-term evolution. A cluster of invariant residues were located on one side of the *β*-stranded “core” domain that is important for molecular interactions [1]. Thaw et al. found that the “core” domain displays significant similarity to that of the Mss4/Dss4 protein family upon analyzing the solution structure of TCTP from *Schizosaccharomyces pombe* [1, 9]. TCTP is also a novel small molecular weight (23 kDa) heat shock protein that protects cells from thermal shock by functioning as a molecular chaperone [10].

### 2.3. Transcriptional and Translational Regulation

Previous reports demonstrated that expression of TCTP is regulated at the level of the transcription as well as the translation [2, 11]. TCTP is translationally regulated: abundant TCTP mRNA was found as untranslated mRNP particles [11], an increase in TCTP synthesis under the treatment of transcription inhibitor, actinomycin D [12], a 5′-TOP of TCTP mRNA and its extended secondary structure [8].

Comparing the promoter region of TCTP in human, mouse, rat, rabbit, and dog predicted the transcription factor binding sites of TCTP. Not surprisingly, the promoter regions of TCTP are also highly conserved among these species. Andree et al. further demonstrated that the transcription of TCTP is controlled by cAMP signaling via phosphorylation-dependent activation of CRE/CREB interaction [7]. In our previous study, we utilized chromatin immunoprecipitation-based (ChIP-based) cloning strategy to identify genes potentially regulated by CHD1L (chromodomain helicase/ATPase DNA binding protein 1-like gene) [13]. From this strategy, we isolated 35 CHD1L-binidng loci and characterized a specific CHD1L-binding motif (C/A-C-A/T-T-T-T). Two CHD1L-binding motifs have been identified at −748 bp and −851 bp in the 5′-flanking region of TCTP [13]. Importantly, we have demonstrated that the binding of CHD1L to the promoter region of TCTP dramatically activates the transcription of TCTP.

## 3. Biological Functions

### 3.1. Growth and Development

A knockout mouse approach had been used to investigate the role of TCTP in development. As demonstrated by Chen et al., heterozygous mice (TCTP^+/−^) were viable, fertile, and morphologically similar to their wild-type littermates, while the homozygous (TCTP^−/−^) were embryonic lethal. Moreover, TCTP^−/−^ embryo at embryonic stage day 5.5 (E5.5) suffered from reduced cell numbers and increased apoptosis and subsequently died around E9.5–10.5 [14]. This suggests that TCTP is essential for normal development. The human TCTP (hTCTP) protein sequence is 50% identical with* Drosophila* TCTP (dTCTP) [15]. They also indicated that silencing of dTCTP by RNA interference resulted in the reduced cell size, cell number, and organ size. Also, as Rheb is an important regulator in TSC-mTOR pathway, TCTP might function as a growth-regulating protein by the stimulation of GDP/GTP exchange of human Rheb (hRheb) via binding to hRheb [15]. On the contrary, Rehmann et al. argues that TCTP does not act as a guanine nucleotide exchange factor (GEF) of Rheb [16]. Therefore, additional experiments are essential to further substantiate the role of TCTP in cell growth regulation through the TSC-mTOR pathway.

### 3.2. Regulation of Cell Cycle and Apoptosis

Gachet et al. demonstrated that TCTP interacts with microtubules during G1, S, G2, and early M phase of the cell cycle [17]. During mitosis, it binds to the mitotic spindle and detaches from the spindle during the metaphase-anaphase transition. When TCTP is overexpressed in bovine mammary epithelial cells, rearrangement of microtubule and growth inhibition can be observed [17]. Two-hybrid screening methods identified TCTP as a substrate of polo-like kinase (Plk), which is involved in the formation and function of bipolar spindles and Plk phosphorylates TCTP on two serine residues [18]. Abolishing Plk phosphorylation on TCTP-induced mitotic defects and high incidences of apoptosis, indicating that phosphorylation of TCTP on two serine residues by Plk plays an important role in cell mitosis [18].

### 3.3. Regulation of Self-Renewal and Pluripotency

Homeodomain transcription factors Oct4 and Nanog have been identified as master regulators of stem cell self-renewal and pluripotency [19]. Oct4 appears to regulate cell fates in a quantitative fashion and maintain a critical concentration to sustain embryonic stem (ES) cell self-renewal [20]. Proteins associated with the regulatory region of the mouse *oct4* gene can be isolated and identified by mass spectrometry [21]. Using this strategy, TCTP was found to bind to the promoter region of *oct4*. Although *tpt1* transcript depletion can inactivate the transcription of both *oct4* and *nanog*, TCTP binds only to the* oct4 *promoter as determined by ChIP analysis in mouse ES cells [21]. It has also been suggested that TCTP could regulate *oct4* by directly binding to its proximal promoter. However, TCTP might also regulate *nanog* indirectly or binds to its distal promoter. These data suggest that TCTP is a potential regulator of self-renewal and pluripotency.

## 4. Malignant Transformation by TCTP during Cancer Progression

Although there are many distinct types of human cancers, six essential alterations to normal cells are believed to define the progression of most human malignancies: they are evasion of apoptosis, sustained angiogenesis, accelerated cell cycle progression, tissue invasion and metastasis, self-sufficiency in growth signals, and insensitivity to antigrowth signals. Therefore, the information described below provides us with some insights into the oncogenic role of TCTP.

### 4.1. Differential Expression of TCTP in Cancer

Independent studies indicated that TCTP is preferentially expressed in cancer. In human colon cancer, the level of TCTP mRNA was detected in three human colon carcinoma cell lines (SNU-C2A, SNU-C4, and SNU-C5). The expression levels were not equal among these cell lines. SNU-C5 with the highest expression grew at the fastest rate; however, SNU-C2A with the lowest expression grew at the slowest rate [4]. Higher expression level of TCTP was also observed in prostate cancer specimens compared to normal prostate tissues [5]. In hepatocellular carcinoma (HCC), the expression level of TCTP was detected in a retrospective cohort of 118 HCC patients. As a result, TCTP was found to be significantly upregulated in tumor tissues when compared to their adjacent noncancerous tissues. Overexpression of TCTP (defined as 2 fold increase) was detected in 40.7% of HCC specimens [6].

### 4.2. Antiapoptosis

Overexpression of TCTP was detected in many types of tumors and its downregulation decreases the viability of those cells [3]. These suggest that TCTP is a prosurvival factor in normal and cancer cells.

Myeloid cell leukemia 1 (Mcl-1) is an antiapoptotic protein identified as an early gene induced during differentiation of ML-1 myeloid leukemia cells. It is a member of Bcl-2 family that plays a pivotal role in animal development. Zhang et al. found that TCTP interacts with Mcl-1, but not any other Bcl-2 family member. Further, the depletion of Mcl-1 rapidly destabilized TCTP in an osteosarcoma cell line U2OS, supporting the conclusion that Mcl-1 serves as a chaperone of TCTP, binding and stabilizing TCTP* in vivo* [22]. On the contrary, Liu et al. suggest that TCTP may also serve as a molecular chaperone and cofactor of Mcl-1, in which the association between TCTP and Mcl-1 is essential for both to function [23].

It has been reported that TCTP interacts with Bcl-xL, an antiapoptotic protein that maintains the integrity of the mitochondrial membrane [24]. They found that the N-terminal region of TCTP is responsible for its interaction with the Bcl-xL BH3 domain, which is critical for eliciting antiapoptotic properties. They also proposed that TCTP might inhibit T-cell apoptosis by preventing the phosphorylation/inactivation of Bcl-xL. More recently, the crystal structure of TCTP provides new insights into its antiapoptotic activity. The H2-H3 helices of TCTP share a structural similarity to the H5-H6 helices of Bax [25]. Mutation of residues (E109 and K102) close to the turn between the two helices H2 and H3 of TCTP reduces the antiapoptotic effect of TCTP on Bax-induced apoptosis, indicating that H2-H3 helices of TCTP play an important role in the inhibition of apoptosis. Despite the lack of evidence to support the binding between TCTP and Bax [22, 23, 25], Susini et al. suggested that the anchorage of TCTP into the mitochondrial membrane could inhibit the dimerization of Bax and subsequent Bax-induced apoptosis.

### 4.3. Mitotic Defects and Chromosome Missegregation

**Figure 1:**
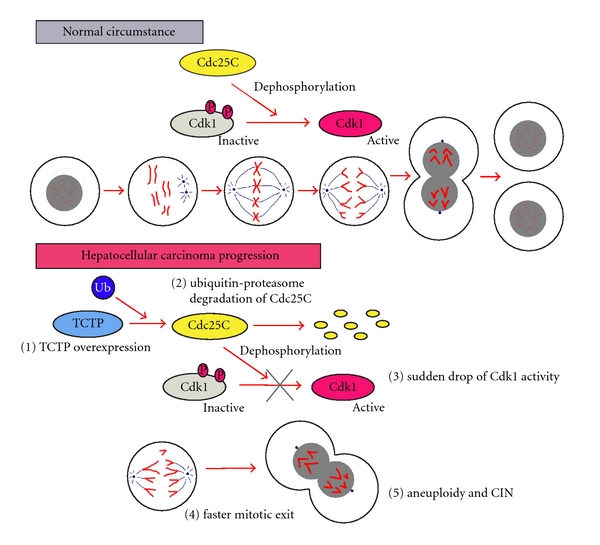
Mechanistic diagram showing the effect of abnormal regulation of TCTP/Cdc25C/Cdk1 pathway in HCC development. (Upper panel) Under the normal mitotic progression, Cdc25C activates Cdk1 by the dephosphorylation of Thr14 and Tyr15 in Cdk1. The level of active Cdk1 is a key factor for maintaining the mitotic state and functions as a key switch for cell division. (Lower panel) During the HCC development, TCTP is overexpressed in over 40% of HCC cases. Overexpression of TCTP promotes the ubiquitin-proteasome degradation of Cdc25C, which leads to the failure in the dephosphorylation of Cdk1 on Tyr15 and decreases Cdk1 activity. As a consequence, the sudden drop of Cdk1 activity in mitosis induces a faster mitosis exit and chromosome missegregation, which leads to aneuploidy and CIN, finally causing cancer development.

In mitosis, APC is activated by binding to Cdc20, and this is dependent on high Cdk1 activity [26]. Subsequently, the active APC recognizes securin and cyclin B, thereby provoking their degradation. Degradation of cyclin B inactivates Cdk1, which subsequently permits mitotic exit [27]. The microtubule binding activity and Plk phosphorylation sites indicate that TCTP is an important gene in the regulation of mitotic progression [17, 18]. As reported in our previous study, the role of TCTP in cell cycle progression has been fully investigated by overexpressing TCTP in HCC cell lines. As a result, TCTP has no obvious effect on G1/S transition; however, when cells were released after synchronization at the prometaphase, an accelerated mitotic exit was observed in TCTP-overexpressing cells [6]. Mechanistic study demonstrated that TCTP promoted the ubiquitin-proteasome degradation of Cdc25C during mitotic progression, which caused the failure in the dephosphorylation of Cdk1-Tyr15 and decreased Cdk1 activity. As a consequence, the sudden drop of Cdk1 activity in mitosis induced a faster mitotic exit and chromosome missegregation, which led to chromosomal instability (Figure 1). We did not observe any obvious difference in cyclin B1 expression level between control and TCTP overexpressing cells, suggesting that the TCTP-mediated faster mitotic exit might not be related to APC-mediated degradation of cyclin B1. Xenograft experiments further supported our notion that the overexpression of TCTP could induce mitotic defects and chromosome missegregation [6].

### 4.4. Migration and Metastasis

Metastasis is the final step in solid tumor progression and is the most common cause of death in cancer patients [28]. Metastasis is a multistep process, all of which must be successfully completed before giving rise to a metastatic tumor. It has been reported that TCTP is preferentially expressed in colon cancer cell lines (LoVo, SW620) with highly metastatic potentials. Depletion of TCTP by shncRNA-TCTP in LoVo cells significantly reduced the number of the hepatic surface metastases in nude mice [29]. Recently, our group has studied the motile and invasive capabilities of TCTP *in vitro* and *in vivo*. As a result, the number of invaded cells was significantly increased in TCTP-overexpressing cells. An experimental metastasis assay was used to examine the metastatic nodules formed in the livers of SCID mice after inoculation with TCTP-overexpressing cells. Cells were injected through the tail-vein of SCID mice, metastatic nodules were counted at 8 weeks after injection. The number of metastatic nodules on the surface of the liver was significantly higher in mice injected with TCTP-transfected cells.

## 5. Clinical Implication of TCTP in Human Cancers

Overexpression of TCTP was found in different types of cancers, including colon cancer [4], prostate cancer [5], and liver cancer [6]. In our previous study, overexpression of TCPT was detected in 40.7% (48 of 118) of HCC cases. Clinically, overexpression of TCTP was significantly associated with the advanced tumor stage and overall survival time of HCC patients. TCTP was also determined as an independent marker associated with poor prognostic outcomes [6]. Moreover, our recent study also indicated that the overexpression of TCTP was significantly associated withextrahepatic metastases (e.g., bone, lymph node, and kidney) among HCC patients.

By comparing the proteome of a melanoma cell line (MeWo) and their chemoresistant counterpart, TCTP was also found to be one of the proteins preferentially expressed in chemoresistant melanoma cell lines [30].

## 6. Conclusions and Future Directions

Due to the ubiquitous expression and high-degree conservation, TCTP protein underlines its important functions in the cell. An increasing number of research investigations are being conducted in this area, particularly into the effect of TCTP during cancer progression. It is implicated in cell growth, cell cycle progression, apoptosis, and regulation in pluripotency. Although TCTP is not tumor-specific, preferential expression of TCTP in different types of cancer underlines the importance of TCTP in cancer progression. As summarized in this paper, TCTP mainly exerts its tumorigenic function via inhibiting apoptosis, accelerating mitotic exit, inducing invasion and metastasis, and so on. By using molecular biological techniques, we demonstrated a molecular pathway, TCTP/Cdc25c/Cdk1, which plays an important role in hepatocarcinogenesis by accelerating mitotic progression and inducing CIN (Figure 1). CIN is a hallmark of many types of human cancers and is significantly associated with poor prognosis. Thus, characterization of this novel pathway will greatly facilitate our insights into the link between aneuploidy cancer development. To better understand the oncogenic mechanism of TCTP, our current work is focusing on the DNA-binding activity of TCTP and identifying its specific binding motifs. Future research on the regulatory network of TCTP will improve our understanding of this oncogene and may ultimately contribute to the development of more accurate treatment modalities.
